# Distance and Microsphere Aggregation-Based DNA Detection in a Paper-Based Microfluidic Device

**DOI:** 10.1177/2472630319887680

**Published:** 2019-11-13

**Authors:** Brent Kalish, Jianhou Zhang, Hilary Edema, James Luong, Jenna Roper, Chad Beaudette, Richard Echodu, Hideaki Tsutsui

**Affiliations:** 1 Department of Mechanical Engineering, University of California Riverside, Riverside, CA, USA; 2 Gulu University Bioscience Research Laboratories, Gulu, Uganda; 3 Department of Bioengineering, University of California Riverside, Riverside, CA, USA; 4 Department of Biology, Faculty of Science, Gulu University, Gulu, Uganda

**Keywords:** distance-based, microsphere, aggregation, DNA detection, paper-based

## Abstract

In paper-based microfluidics, the simplest devices are colorimetric, giving qualitative results. However, getting quantitative data can be quite a bit more difficult. Distance-based devices provide a user-friendly means of obtaining quantitative data without the need for any additional equipment, simply by using an included ruler or calibrated markings. This article details the development of a quantitative DNA detection device that utilizes the aggregation of polystyrene microspheres to affect the distance that microspheres wick through filter paper. The microspheres are conjugated to single-stranded DNA (ssDNA) oligomers that are partially complementary to a target strand and, in the presence of the target strand, form a three-strand complex, resulting in the formation of aggregates. The higher the concentration of the target strand, the larger the aggregate, and the shorter the distance wicked by the microspheres. This behavior was investigated across a wide range of target concentrations and under different incubation times to understand aggregate formation. The device was then used to successfully detect a target strand spiked in extracted plant DNA.

## Introduction

Recently, paper-based microfluidics have attracted interest for their promise of providing low-cost point-of-care diagnostics. Paper-based devices are low-cost, lightweight, and often very user-friendly, ideal attributes for use in resource-limited settings and by those without extensive technical training. One of the most common types is lateral flow devices, which generally only provide qualitative or semiquantitative results.^1,2^ Many existing quantitative devices require cameras to determine color intensity,^3,4^ or multimeters to determine current^5^ or resistance^6^ changes, from which analyte concentrations can be determined. Distance-based methods, however, have been proposed to be the ideal solution for providing instrument-free, quantitative results.^7–9^ These methods often utilize a color-changing reaction that occurs as fluid wicks along a channel, where the length of the colored region corresponds to the concentration of the targeted analyte.^9–16^ The specific reaction used depends on the target analyte, requiring each new device to be designed for a different suitable reaction. Other distance-based methods utilize other mechanisms, such as fluid viscosity^17^ or blood coagulation,^18^ affecting wicking speeds. Here we build upon our previous work^19^ and investigate the process of aggregate formation and the viability of detecting targeted DNA in extracted plant material.

**Figure 1.**
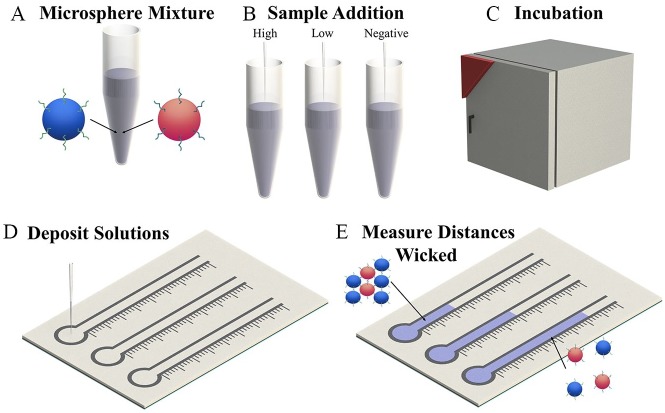
Process flow of the proposed microsphere aggregation and distance-based ssDNA detection scheme. (**A**) Microspheres conjugated to two different noncomplementary oligomers are mixed together. (**B**) Samples containing the targeted strand are added to the microsphere mixture. (**C**) The microsphere solution is incubated at 45 °C for 30 min. (**D**) The microsphere solution is deposited in the channel inlet, where channels have been defined using solid wax printing. (**E**) The microspheres in solutions containing the target strand have aggregated, resulting in reduced wicking distances.

The mechanism behind this detection method is the target-induced aggregation of two populations of microspheres, each conjugated to noncomplementary DNA oligomer probes (**Fig. 1**). The probes are each partially complementary to the target analyte. Upon addition of the target to a mixture of the microspheres, they begin to aggregate. Unaggregated, the microspheres are small enough to wick through the paper substrate unimpeded, but when aggregated, there are fewer available microspheres to wick through the paper and the aggregates themselves are too large to wick through the paper’s pores. The degree of aggregation directly affects the distance wicked, with larger aggregates (caused by higher analyte concentrations) resulting in shorter wicked distances.

## Materials and Methods

### Paper-Based Microfluidic Device Fabrication

The device was patterned in SolidWorks (Dassault Systèmes, Vélizy-Villacoublay, France) and printed onto Whatman grade 4 filter paper (GE Life Sciences, Pittsburgh, PA) using a solid wax ink printer (ColorQube 8880; Xerox, Norwalk, CT). The wax was then melted in an oven (FD 53; Binder, Tuttlingen, Germany) at 170 °C for 2 min, allowing it to penetrate the thickness of the paper. The paper was then run through the printer again, printing a solid layer of wax across the bottom side. The bottom of the paper was then sealed using packing tape to prevent leakages. The ruler markings have 2 mm spacing. **Supplemental Figure S1** depicts the device’s layered structure.

### Conjugation of ssDNA to Microspheres

Stock 10% solid 1 µm polystyrene latex carboxylated microspheres (Magsphere, Pasadena, CA) were washed 3× in 10 mM 2-(*N*-morpholino) ethanesulfonic acid (MES) and diluted to 3% solid. The microsphere solution was then mixed with one of the single-stranded DNA (ssDNA) probes (probe A or probe B) at a 1:4 probe-to-surface carboxyl ratio and excess 1-ethyl-3-(3-dimethylaminopropyl) carbodiimide (EDC) and *N*-hydroxysuccinimide (NHS). After a 30 min incubation, additional EDC and NHS were added before another 30 min incubation period. Afterward, the microsphere solution was washed once in 1% Tween-20 in 10 mM phosphate-buffered saline (PBS), returned to 3% solid, and then washed 2× in 10 mM PBS. The sequences of all strands used in this study are listed in **Table 1**.^20^ All chemicals were purchased from Sigma-Aldrich (St. Louis, MO) and DNA was purchased from Integrated DNA Technologies (Coralville, IA).

**Table 1.** Probe and Target Sequences.

Name	Sequence
Probe A^20^	5′-/5AmMC6//iSp18/TTT TTT TTT TCG CAT TCA GGA T-3′
Probe B^20^	5′-TCT CAA CTC GTA TTT TTT TTT T/iSp18//3AmMC7/-3′
A′-B′^20^	5′-TAC GAG TTG AGA ATC CTG AAT GCG-3′
Strand C	5′-CCG TGG TAG TGT ATC CTG AAT GCG-3′
Strand D	5′-CCG TGG TAG TGT CAG TGT CGT GTT-3′

Strands C and D were generated randomly for this study.

### DNA Extraction from Plant Leaves

DNA was extracted from sour orange (*Citrus* × *aurantium*) leaves using Plant DNAzol (Thermo Fisher Scientific, Waltham, MA) according to the manufacturer’s recommended protocol (**Suppl. Fig. S2**). Briefly, leaf tissue was homogenized using a mortar and pestle. Homogenized tissue was then mixed with DNAzol and chloroform before centrifuging at 12,000*g* for 15 min. The upper aqueous phase was then removed and DNA was precipitated using 100% ethanol. The DNA was pelleted via centrifugation at 5000*g* for 4 min and then washed with a 1:0.75 DNAzol–ethanol solution, followed by a wash with 75% ethanol. The DNA was then dissolved using 8 mM NaOH (100 µL/200 mg of leaf tissue) and neutralized using HEPES buffer. The DNA concentration of the extracts ranged from 90 to 130 ng/µL. Extracts were spiked with our target strand, A′-B′, before or after the extraction process. All chemicals were purchased from Sigma-Aldrich (St. Louis, MO).

### Wicking Test Protocol

Microsphere solutions (30 µL, 2% solid) comprising equal parts probe A-conjugated microspheres, probe B-conjugated microspheres, and target strand (A′-B′) were incubated for 30 min at 45 °C to promote hybridization and then deposited in the inlets of 2 mm wide wax-printed paper-based microfluidic channels. The channels were left to wick for approximately 15 min in a humidity chamber (Model 5503; Electro-Tech Systems, Glenside, PA) kept at 55% relative humidity and 23 °C. For ease of differentiating which probe was conjugated to which set of microspheres, red and blue microspheres were used. For the fluorescent tests, red and green fluorescent microspheres were used instead.

### Aggregate Size Analysis

Custom 1 µL wells were constructed using laser-cut 0.5 mil polyamide tape (Caplinq, Orléans, ON, Canada) stuck to glass microscope slides. Wells were capped with an ultrathin (0.085–0.115 mm) coverslip (Thorlabs, Newton, NJ) and observed under 100× magnification using a DM2000 fluorescent microscope (Leica Microsystems, Wetzlar, Germany). The aggregate observation wells are depicted in **Supplemental Figure S3**.

## Results and Discussion

### Size-Based Wicking

**Figure 2.**
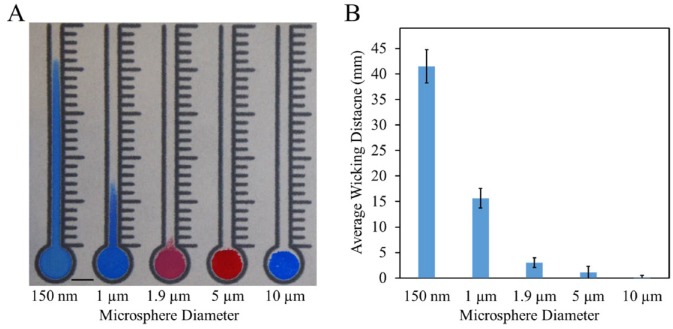
Size-based wicking. (**A**) Wicking distances of 2% solid microsphere solutions in the paper channels. Microsphere solution (30 µL) was added to the center of each inlet. Scale bar is 5 mm. (**B**) Average wicking distances measured from the top of the inlet. Data are displayed as mean ± standard deviation (*N* = 20).

The Whatman grade 4 filter paper chosen for these experiments is described as having a particle retention size of 22–25 µm. This is a descriptor of a filter paper’s filtration and does not correspond to the sizes of particles capable of lateral wicking. **Figure 2** depicts the relative difference in wicking distances of solutions containing 2% solid microspheres of different sizes. The smallest microspheres, 150 nm, traveled the farthest, more than 40 mm, while the largest microspheres, 10 µm, were too large to wick at all and sat on the surface of the paper. The 1 µm microspheres were chosen for subsequent experiments, as preliminary studies found that the 150 nm microspheres did not readily form aggregates large enough to significantly change their overall wicking distance.

### Microsphere Concentration

**Figure 3.**
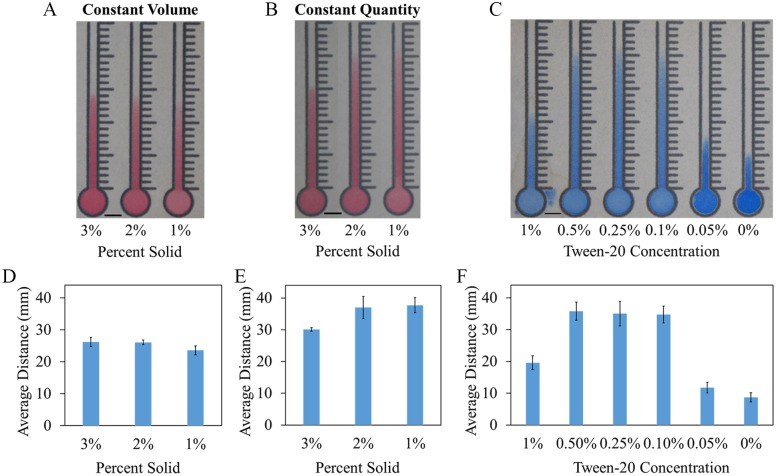
Microsphere solution optimizations. (**A**) Constant volume. Wicking distances of 1%–3% solid microspheres in the paper channels when the volume liquid deposited in each channel is kept constant (30 µL). (**B**) Constant quantity. Wicking distances of 1%–3% solid microspheres in the paper channels when the quantity of microspheres in each channel is kept constant. (**C**) Surfactant optimization. Wicking distances of 2% solid microspheres in Whatman grade 4 filter paper in solutions containing different Tween-20 concentrations. (**D**) Constant volume average wicking distances measured from the top of the inlet (*N* = 6). (**E**) Constant quantity average wicking distances measured from the top of the inlet (*N* = 4). (**F**) Surfactant optimization average wicking distances measured from the top of the inlet (*N* = 10). Data are displayed as mean ± standard deviation. Scale bars are 5 mm.

In order to determine how wicking distances were affected by microsphere concentration, two experiments were run using the 1 µm microspheres. The first maintained a constant volume (30 µL) with varying concentrations, while the second kept the number of microspheres constant as the concentration (and overall solution volume) changed. Solutions containing 1%, 2%, and 3% solid microspheres were tested. When volumes were held constant, decreasing microsphere concentration resulted in channels that were paler and wicked slightly shorter distances (**Fig. 3A**); however, when microsphere quantities were held constant, as the concentration of microspheres decreased (and volume increased), the distances wicked increased (**Fig. 3B**). As shown in **Figure 3D,E**, the actual volume of liquid used seems to play a larger role in wicking distances than the actual quantity of microspheres. Larger volumes (and corresponding lower microsphere concentrations) were tested (not shown), but almost universally leaked, as their volumes exceeded the channels’ capacity. This excess liquid eventually leaked through the unmelted wax barrier at the bottom of the channel and slipped under the melted wax walls.

The high dependence of the microspheres’ overall wicking distances on the deposited volume (**Fig. 3B,E**) suggests that the sensor’s performance will be highly susceptible to variations in ambient relative humidity and temperature, as low relative humidity and high temperature can result in considerable evaporation of the deposited liquid before it has wicked the full distance through the paper. Evaporation will not only result in a reduction in volume of the wicking fluid, but also increase the concentration of the microspheres, further compounding a reduction in overall wicking distances. All wicking experiments were performed in a humidity chamber kept at a constant relative humidity (55%) and temperature (23 °C); however, for use outside a lab, care will need to be taken to seal the sensor to eliminate any variation based on the user’s ambient environmental conditions.

### Surfactant Concentration

The presence of small amounts of a surfactant can significantly increase the distance wicked by the microspheres. At high concentrations (>1%) of Tween-20, microsphere solutions were able to penetrate the wax boundaries of the channel, resulting in dramatically reduced wicking distances. Solutions containing low (<0.1%) or no surfactant resulted in microspheres that did not travel very far. However, within those bounds, the microspheres are able to wick much farther (**Fig. 3C**). In all subsequent experiments, a Tween-20 concentration of 0.1% was used to minimize the potential for leakage while maximizing wicking distances.

### Target Concentration-Dependent Wicking

**Figure 4.**
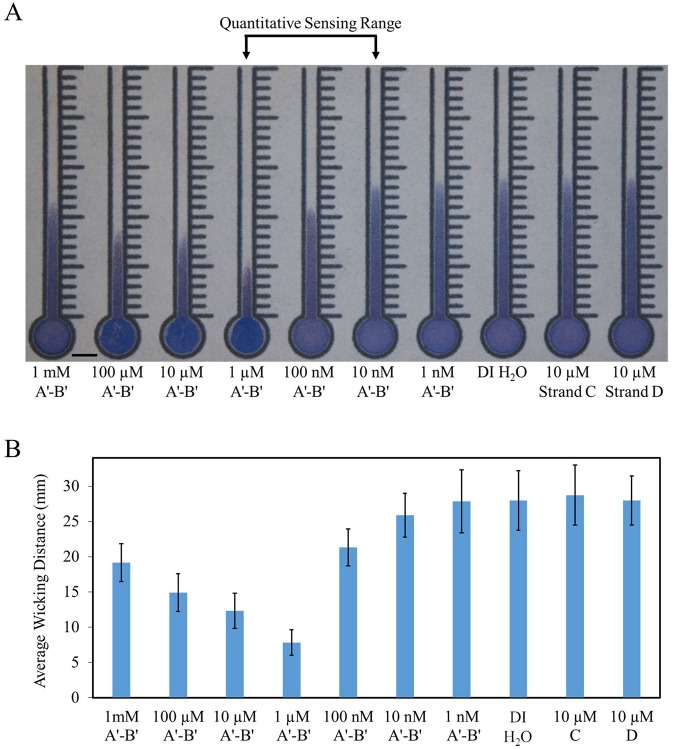
Target concentration-dependent wicking. (**A**) Wicking distances of 2% solid microsphere solutions in the paper channels. Seven different A′-B′ linker concentrations were tested, ranging from 1 nM to 1 mM, along with a DI H_2_O control, and 10 µM strands C and D. A′-B′ is partially complementary to both probes A and B, while strand C is partially complementary to only probe A and strand D is not complementary to either probe. Thirty microliters of 2% solid microsphere solution (equal parts strand A-conjugated microspheres, strand B-conjugated microspheres, and linker strand) deposited in the inlet of each channel after 30 min of incubation at 45 °C. Scale bar is 5 mm. (**B**) Average wicking distances measured from the top of the inlet. Data are displayed as mean ± standard deviation (*N* = 30). (Two conditions, 1 µM and 10 nM, are *N* = 29.)

As a demonstration of the viability of this detection mechanism to quantify ssDNA, microspheres conjugated with probe A and probe B were mixed with a target strand, an oligomer partially complementary to both probes (A′-B′), and were then incubated for 30 min at 45 °C before being deposited into channels made on Whatman grade 4 filter paper (**Fig. 4**). At lower target concentrations (10 nM–1 µM), the distance wicked by the microspheres was inversely proportional to the target concentration (shorter distances at higher concentrations), while at higher concentrations (>1 µM), the distance traveled was proportional to the target concentration (longer distances at higher concentrations). When mixed with oligomers only complementary to one (strand C) or neither (strand D) probe, the microspheres failed to aggregate and displayed nearly identical wicking behavior to microspheres mixed with only H_2_O, suggesting that the aggregation is hybridization induced. The overlapping standard deviations found at the low- and zero-target concentrations are a result of the overall wicking distances for those conditions being near the maximum possible wicking distance for that concentration and quantity of microspheres. As a result, the slight differences in wicking distances caused by variations in deposited volumes are more apparent. The paper-to-paper variation also contributes to the large standard deviations of those conditions. To understand the extent of this effect, the wicking distances of each channel on each sheet were normalized to the distance wicked by their respective control channel, deionized (DI) H_2_O (**Suppl. Fig. S4**). This normalization resulted in a decrease of the relative standard deviations of all but the 1 mM concentrations by at least 25%.

The inversely proportional range (10 nM–1 µM) is considered the quantitative sensing range, as the difference in distances traveled by the microspheres at each target strand concentration is much larger than that of those in the proportional region, as shown in **Figure 4**. The proportional wicking behavior at high linker concentrations is thought to be caused by excess target strands hybridizing to each available probe, preventing aggregates from forming (**Suppl. Fig. S5**).

For samples with a high concentration of the targeted strand, that the sample is in this range is indicated by the presence of aggregates on the surface of the paper in the channel’s inlet (**Fig. 4A**), although it appears that at extremely high concentrations this effect tapers off, likely due to the rapid and overwhelming saturation of target strands onto the available probes. As such, serial dilutions may be required to both identify which region the sample is in and lower the target concentration into the inversely proportional detection range for subsequent detection.

### Aggregate Formation

**Figure 5.**
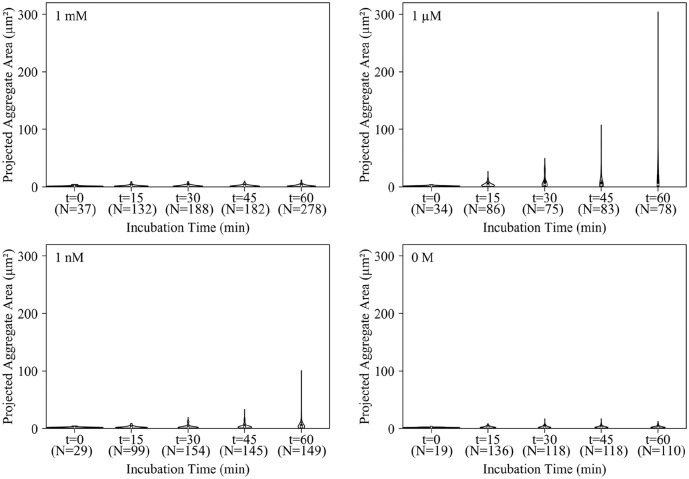
Aggregate size (projected area) distributions of microspheres mixed with 1 mM, 1 µM, 1 nM, and 0 M A′-B′ with varying incubation times in 15 min intervals. Five images for each time point and each concentration were analyzed. Aggregate counts (*N*) for each condition are listed below the corresponding time point. The counts for the *t* = 0 conditions were low due to the microspheres being highly dispersed, and nearly all appeared as individual microspheres.

In order to optimize the incubation step, a thorough understanding of microsphere aggregate formation is needed. To this end, microsphere mixtures were prepared as described above and then, at various time intervals, 1 µL samples were withdrawn and deposited into the aggregate observation wells and observed under a microscope. The size and growth of the microsphere aggregates are displayed in a series of violin plots in **Figure 5**. In these violin plots, the *x* axis of the violin plot is time and the *y* axis shows the projected area of the aggregates. The width of each violin represents the probability density of that size. The interquartile range is indicated inside each violin as well.

The microspheres mixed with 1 nM target formed aggregates gradually, and after 60 min a large fraction of the aggregate population remained small. This matches the previous wicking data that showed microspheres mixed with 1 nM target wicked the same distance as the 0 M control. Microspheres mixed with 1 µM target, on the other hand, formed aggregates immediately, and by 60 min nearly all of the microspheres were bound in aggregates. The largest aggregates were more than 300 µm^2^. The 1 mM target microspheres formed rather small aggregates, and the overall size and distribution did not change after 15 min. This seems to support the explanation that high target concentrations rapidly saturate microsphere surfaces with target strands, allowing only a short time interval for aggregate formation (**Suppl. Fig. S5**).

**Figure 6.**
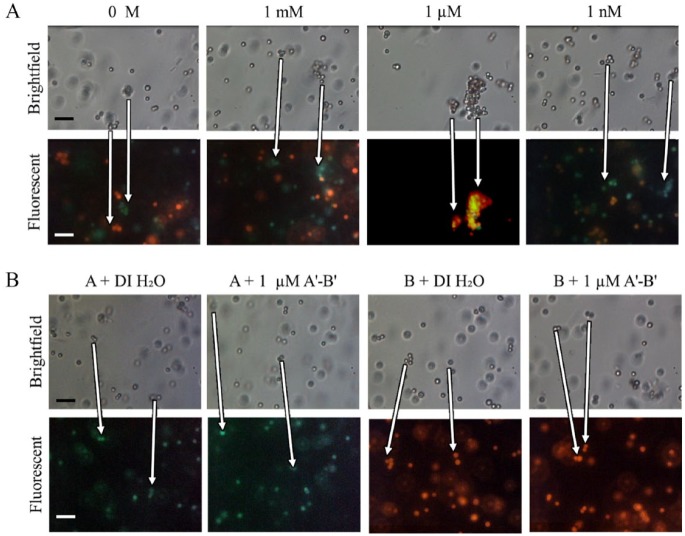
Microsphere aggregate composition. (**A**) Brightfield and composite fluorescent images of 1 µm red and green microspheres mixed with 1 mM, 1 µM, 1 nM, and 0 M A′-B′ after 60 min of incubation. (**B**) Brightfield and fluorescent images of single-color microspheres mixed with either 1 µM A′-B′ or DI H_2_O after 60 min of incubation. Scale bars are 5 µm. Arrows indicate the movement of individual aggregates during the time interval between the brightfield and fluorescent images.

To confirm that the microspheres were hybridizing with their complementary microspheres, aggregates made with red and green fluorescent microspheres were examined under the microscope. **Figure 6A** depicts the aggregates of fluorescent microspheres with brightfield and composite red/green fluorescent filters for 1 mM, 1 µM, 1 nM, and 0 M target concentrations.

In order to verify that the large aggregates only form from mixtures of the two populations of microspheres, each probe was tested separately. The single strands were first tested with DI water, and then tested with 1 µM A′-B′ (**Fig. 6B**). While some small aggregates did form, the aggregates comprised few microspheres and looked broadly similar to the microspheres mixed with DI H_2_O.

### Spiked Plant Extract Detection

#### Detection of Target ssDNA in Postextraction Spike

**Figure 7.**
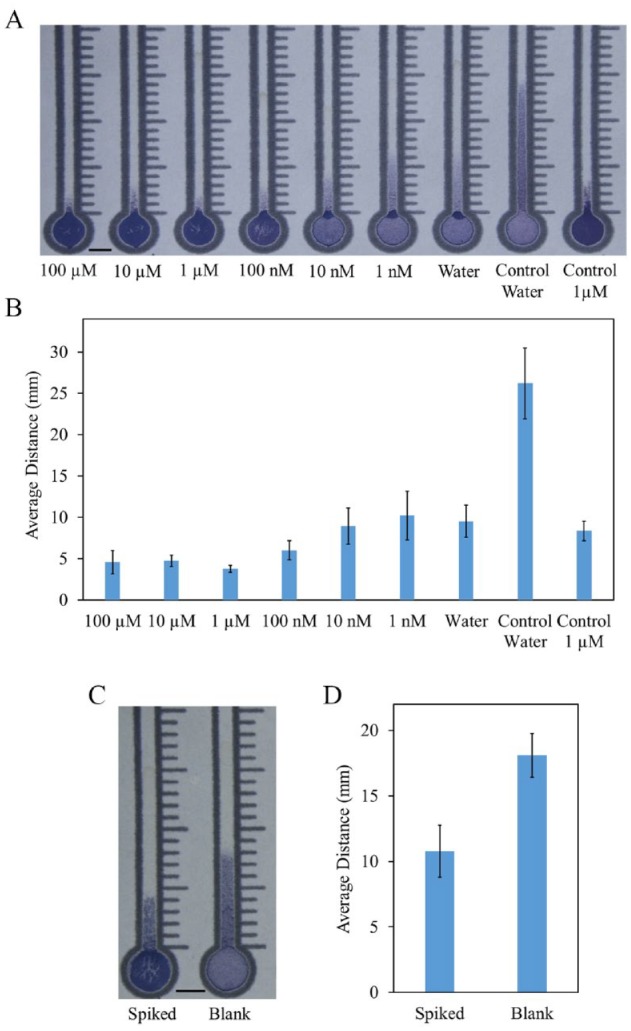
Target concentration-dependent wicking with extracted plant DNA. (**A**) Wicking distances of 2% solid microsphere solutions in the paper channels mixed with extracted plant DNA. DNA extracts from sour orange leaves were spiked with six different A′-B′ linker concentrations, ranging from 1 nM to 100 µM, along with a DI H_2_O control. Two more controls were tested with microspheres mixed with 1 µM and 0 M A′-B′ in water. (**B**) Average wicking distances measured from the top of the inlet (*N* = 4). (**C**) Wicking distances of 2% solid microsphere solutions in the paper channels mixed with extracted plant DNA. Sour orange leaves were spiked with 10 µL of 10 µM A′-B′ before extraction, resulting in a nominal postextraction concentration of 1 µM. (**D**) Average wicking distances measured from the top of the inlet (*N* = 3). Data are displayed as mean ± standard deviation. Scale bars are 5 mm.

Aliquots of DNA extracted from the sour orange leaves were spiked with A′-B′, resulting in concentrations ranging from 1 nM to 100 µM. These solutions were then mixed with the microspheres as described above. The wicking behavior of the spiked mixtures performed broadly similar to those mixed with A′-B′ in water, but with overall shorter wicking distances (**Fig. 7A,B**). The shorter distances are likely due to the higher viscosity of the resolubilized DNA and other compounds that were precipitated out with the DNA during the extraction process. Diluting the extracts would likely solve this issue, but it may reduce sensitivity. A brief example of this is depicted in **Supplemental Figure S6**.

#### Detection of Target ssDNA in Preextraction Spike

As a proof of concept of detecting a DNA strand present in the leaf sample before extraction, ground leaf tissue was spiked with A′-B′ before the extraction, enough to result in a nominal concentration of 1 µM. As depicted in **Figure 7C,D**, the microsphere mixture mixed with the spiked extract wicked a shorter distance than the unspiked control. While the distances traveled by the spiked sample do not match the distance traveled by the 1 µM postextraction spiked microspheres, the extraction process is not lossless. The distance traveled does seem to correspond, though, to an A′-B′ concentration of between 10 and 100 nM.

## Conclusions

In conclusion, microspheres, when conjugated to appropriately complementary probes, rapidly form aggregates in the presence of the target. By depositing these aggregates onto paper, the degree of aggregation can be quantified via the overall distance traveled by the microspheres. Larger aggregates, formed in relatively high concentrations of the target strand, result in the shortest wicking distances, due to the inability of the large aggregates to move through the paper’s pores and due to an overall reduction in the quantity of discrete microsphere particles in solution. This length can then be calibrated to determine the concentration of the targeted DNA strand.

In this work, we have successfully demonstrated the quantitative, distance-based detection of ssDNA in buffer, as well as in extracted plant DNA spiked after extraction with a target ssDNA strand in a paper-based microfluidic device. Further, we demonstrated the qualitative detection of a target ssDNA strand added to plant tissue before DNA extraction. With further calibration, quantitative detection will also be possible.

The plant DNA extraction process used in the above experiments is not very simple or user-friendly, as it requires repeated centrifugation steps and careful removal of certain supernatants. Several groups have worked to develop simpler nucleic acid extraction procedures^21–23^ that enable an untrained user to perform the complete assay. Our lab is also currently developing methods to simplify the extraction procedure.

The direct intended application of this is to detect viral plant pathogens, in particular the sweet potato viruses sweet potato feathery mottle virus and sweet potato chlorotic stunt virus, two viruses that cause a coinfection resulting in the devastating sweet potato virus disease. Both viruses are ssRNA viruses, targets well suited for the proposed microsphere aggregation method. Beyond nucleic acids, microsphere aggregation is likely adaptable to a wide variety of potential analytes, so long as the targets support multiple binding sites.

While the device as detailed above has a detection range from 1 nM to 1 µM, **Supplemental Figure S7** suggests that varying incubation temperatures and cooling strategies may provide for an expanded detection range. For extending the detection range to lower concentrations, amplification will likely be required. The microsphere aggregation method as currently described requires a single-stranded output from an amplification process, which means that many of the common amplification techniques, like PCR or loop mediated isothermal amplification (LAMP), will not work, as they produce double-stranded DNA (dsDNA). Some methods do exist for single-stranded output, such as exponential amplification reaction (EXPAR), which results in ssDNA output,^24,25^ and nucleic acid sequence-based amplification (NASBA), which results in ssRNA output,^26^ and there are some nonenzymatic amplification techniques such as autocatalytic DNA amplification.^27^ It may be possible to adapt the probes to be made from other materials, such as peptide nucleic acids (PNAs) that can insert themselves into the ends of dsDNA,^28^ causing the required aggregation to occur.

## Supplemental Material

SLAS_-_Supplemental_Information_002 – Supplemental material for Distance and Microsphere Aggregation-Based DNA Detection in a Paper-Based Microfluidic Device Click here for additional data file. Supplemental material, SLAS_-_Supplemental_Information_002 for Distance and Microsphere Aggregation-Based DNA Detection in a Paper-Based Microfluidic Device by Brent Kalish, Jianhou Zhang, Hilary Edema, James Luong, Jenna Roper, Chad Beaudette, Richard Echodu and Hideaki Tsutsui in SLAS Technology
