# Supplementary material for: Distance and Microsphere Aggregation-Based DNA Detection in a Paper-Based Microfluidic Device
Source: SLAS Technol. 2019 Nov 13;25(1):58–66. doi: 10.1177/2472630319887680 (PMC6974776; doi:10.1177/2472630319887680)
Supplement: SLAS_-_Supplemental_Information_002 – Supplemental material for Distance and Microsphere Aggregation-Based DNA Detection in a Paper-Based Microfluidic Device [file SLAS_-_Supplemental_Information_002.pdf]

Supplemental Information

Distance and Microsphere Aggregation-Based DNA Detection in a Paper-based Microfluidic Device

Brent Kalish[1], Jianhou Zhang[1], Hilary Edema[2], James Luong[1], Jenna Roper[3], Chad Beaudette[1], Richard Echodu[2,4] and Hideaki Tsutsui[1,3]*

[1]Department of Mechanical Engineering, University of California Riverside, Riverside, CA, 92521, USA
[2]Gulu University Bioscience Research Laboratories, PO Box166, Gulu, Uganda
[3]Department of Bioengineering, University of California Riverside, Riverside, CA, 92521, USA
[4]Department of Biology, Faculty of Science, Gulu University, PO Box166, Gulu, Uganda

*Corresponding Author:
Hideaki Tsutsui, Ph.D.
900 University Ave., Bourns Hall A315
University of California, Riverside
Riverside, CA 92521
Email:htsutsui@engr.ucr.edu

Cross-Section of the Paper-based Microfluidic Device

As shown in Figure S1, the paper-based microfluidic channels used melted wax to form the impermeable boundaries, with an additional printed (but not melted) layer of wax along the bottom. This extra layer, plus the packing tape, ensures that all liquid added to the channel stays in the channel, without leaking out onto the benchtop. The 40 mm x 3 mm channel and 7 mm diameter inlet require approximately 30 µL of liquid to reach the end of the channel.

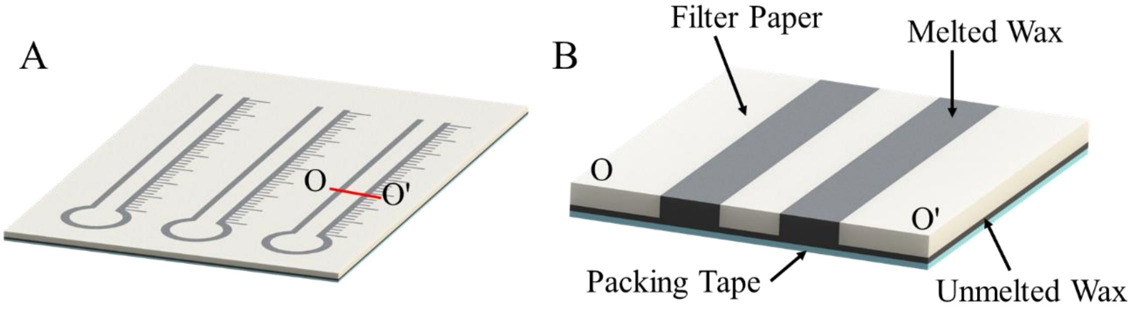

Figure S1. Structure of the assembled device. A) Fabricated device. B) Cross-sectional view.

DNA Extraction Process Flow

Sour orange leaves were ground using a mortar and pestle and then processed using DNAzol according to the protocol described in the DNA Extraction section. An illustration of the process flow is shown in Figure S2.

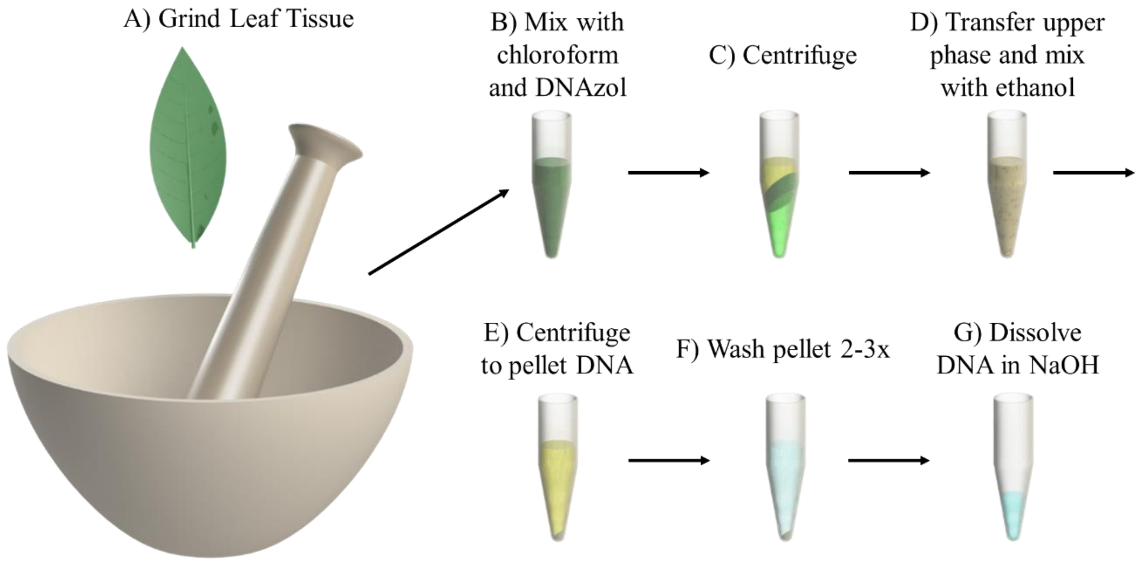

Figure S2. DNA extraction process flow using Plant DNAzol.

Aggregation Observation Well

A well made from laser-cut Kapton tape was used to image free-floating individual aggregates (Fig. S3). An ultra-thin #0 coverslip was required due to the extremely short working distance of the 100x oil immersion objective.

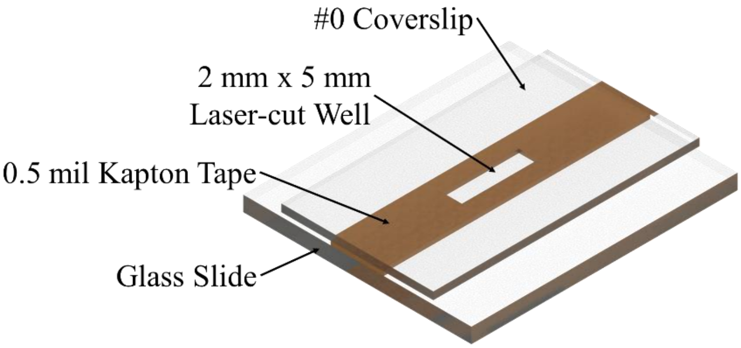

Figure S3. Aggregation observation well.

Normalized Concentration Dependent Wicking Data

The chromatography paper used in the wicking experiments has some variability sheet to sheet, so to take a closer look at the repeatability of the microsphere wicking, each channel on a sheet had its wicking distance normalized to the distance traveled by the DI H_2O channel. The result is that every channel's relative standard deviation fell by at least 25% and up to 65% (except for the 1mM channel, where the relative standard deviation only decreased by 6%).

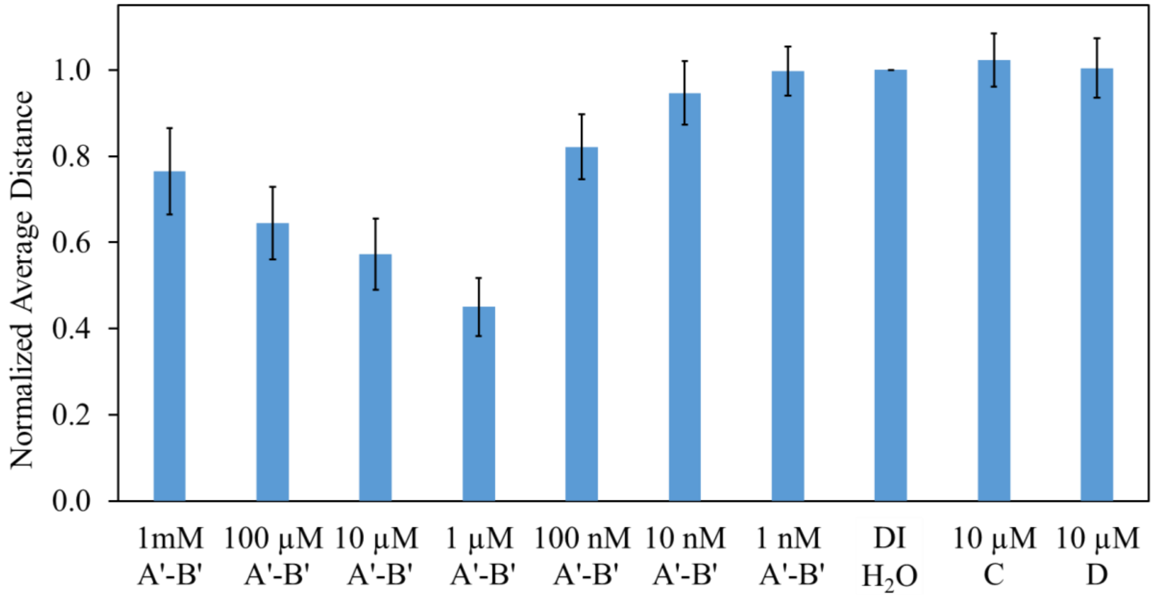

Figure S4. Normalized concentration dependent wicking data. Average normalized wicking distances of the data from Figure 4. Wicking distances in each channel on each sheet were normalized to the length of their respective control channel, DI H_2O. Data is displayed as mean ± standard deviation. N=30. (Two conditions, 1 µM and 10 nM, are N=29)

Hypothesized Mechanism of High Target Concentration Wicking Behavior

As depicted in Figure S4, in the presence of excess target A'-B' each probe (A and B) can be hybridized to a different target strand, resulting in microspheres incapable of forming aggregates. This causes solutions with high target concentrations to wick further than those with medium to low concentrations.

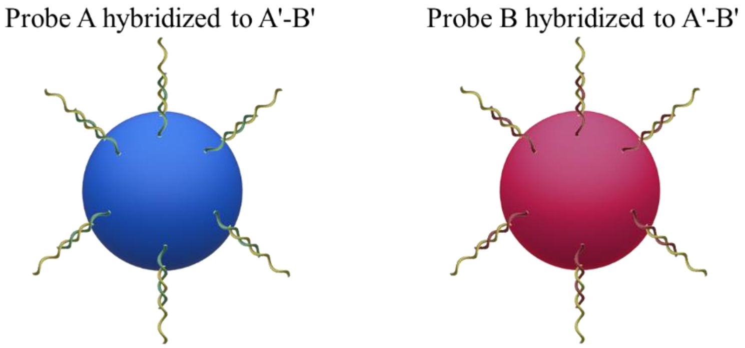

Figure S5. Hypothesized mechanism for high concentration wicking behavior. Target strands (yellow) can bind to every available probe (red and green), preventing 3-strand complexes from forming.

Matrix Effects – Dilution of Pre-Extraction Spiked DNA Extract

Depending on the plant species and the extraction method, there can be a variety of other compounds mixed with the extracted nucleic acid. These constituents make up the sample matrix and their concentrations can have considerable influence on the overall wicking behavior of the microspheres. Additionally, the concentration of the nucleic acid itself may play a role in changing the overall viscosity of the sample added to the microspheres. Figure S6 depicts the wicking distances of microspheres mixed with Pre-Extraction Spiked DNA extract, the same as depicted in Figures 7C and 7D, however, for this test the extracts were diluted to twice the volume, resulting in a nominal concentration of 500 nM in the spiked channel. As a result, the microspheres in the blank channels wicked to approximately the same distance as the DI H_2O controls while the spiked channels wicked to between the 100 nM and 10 nM distances of the buffer tests. As every plant species is different, each assay will need its own optimization or extraction clean up steps to minimize any potential interference with either the wicking of the microspheres through the paper, or the microsphere aggregation process itself.

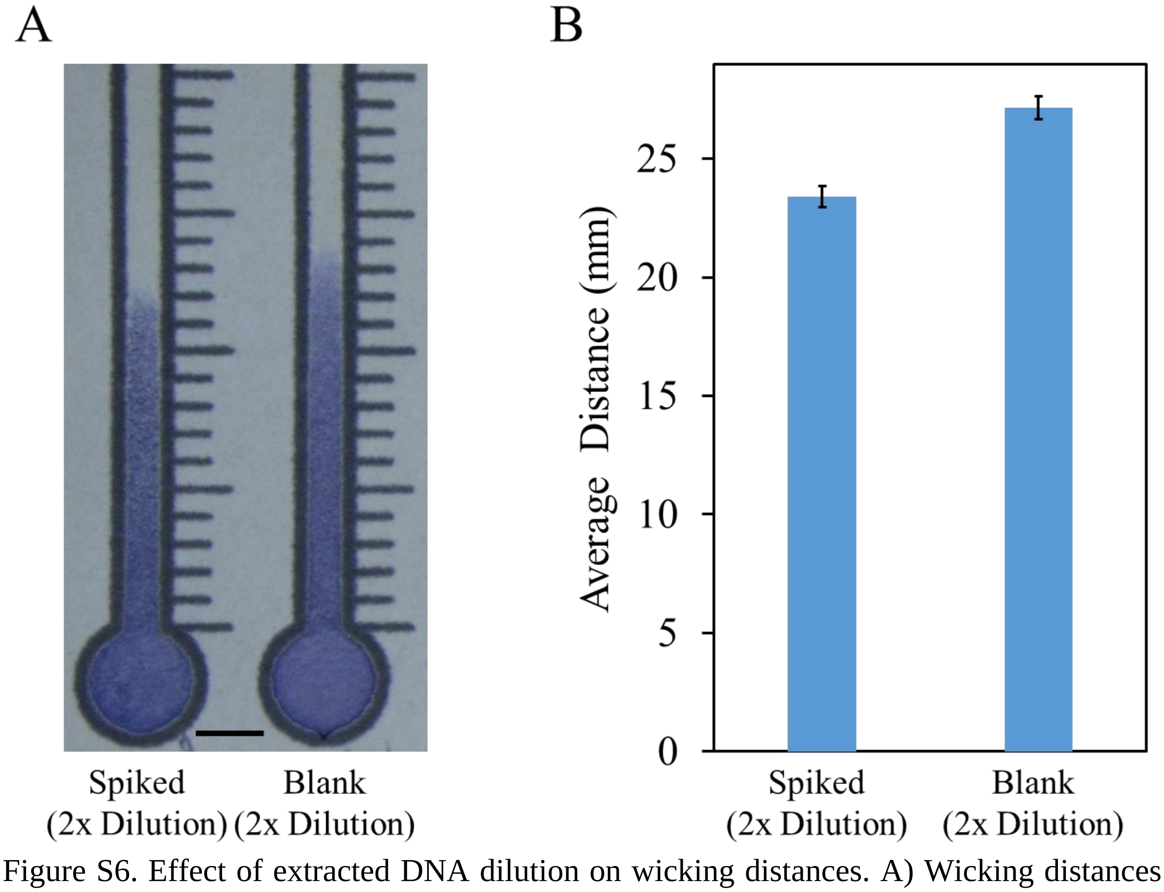

Figure S6. Effect of extracted DNA dilution on wicking distances. A) Wicking distances of 2% solid microsphere solutions in the paper channels mixed with extracted plant DNA. Sour orange leaves were spiked with 10 µL of 10 µM A'-B' before extraction; however, the extracted DNA pellet was resuspended in twice previously tested volume (Figs. 7C and 7D), resulting in a nominal post-extraction concentration of 500 nM. B) Average wicking distances measured from the top of the inlet. N=3. Data is displayed as mean ± standard deviation. Scale bars are 5 mm.

Effects of Elevated Incubation Temperatures

Figure 7 depicts the target concentration dependent wicking behavior of the microspheres in spiked sour orange leaf DNA extract. For that set of experiments, the microspheres were incubated at 45°C, a few degrees below the melting temperature of the probes. However, when incubated at 50°C, the microsphere mixtures did not result in the 1μM channel wicking the shortest distance. Instead, the highest concentrations wicked the shortest distance, preserving the inversely proportional behavior over the entire range, resulting in an expanded detection range (Fig. S7). Further investigation regarding both incubation and cooling times are warranted to maximize sensitivity.

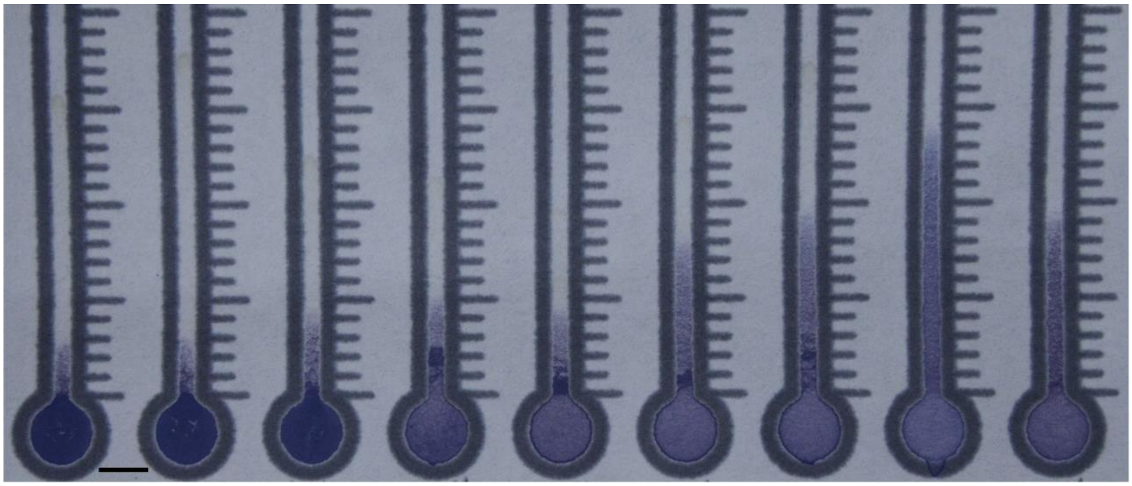

Figure S7. Microspheres mixed with DNA extract from sour orange leaves spiked with A'-B' incubated at 50°C for 30 minutes before deposition. The highest concentrations wicked the shortest distances. The control channels contain microspheres mixed with water or A'-B' in water instead of spiked plant extract. Scale bar is 5 mm.
